# Asymmetric distribution of *cyb-3* in 4-cell stage embryos

**DOI:** 10.17912/W27P4P

**Published:** 2017-03-21

**Authors:** W. Matthew Michael

**Affiliations:** 1 Molecular and Computational Biology Section, Department of Biological Sciences, University of Southern California, Los Angeles, CA 90089, USA

**Figure 1.**
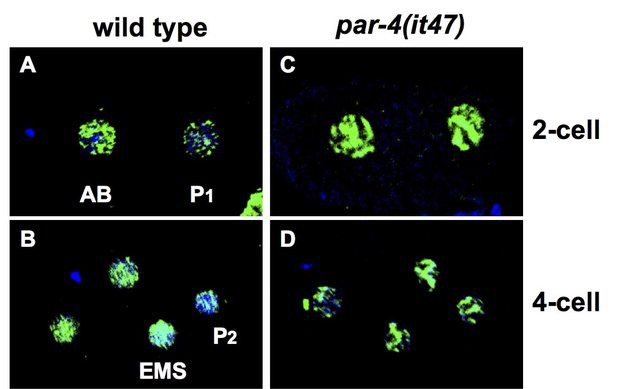


## Description

Early embryos were fixed and stained with Mab F2F4 (green), shown to recognize CYB-3 (Michael, 2016), and DAPI, to illuminate the DNA (blue). Either wild type or *par-4* mutant embryos were examined, after 24-hour incubation at 25C (the non-permissive temperature for the *it47* allele of *par-4*). Anterior is to the left in all images. The data presented here reveals previously not shown data that depicts CYB-3 as asymmetrically distributed at the 4-cell stage. These data further support reported findings in Michael, 2016. There is more CYB-3 in the AB cell relative to its sister P1. In 4-cell embryos there is more CYB-3 in the EMS cell relative to its sister, P2. Thus, during P-lineage divisions, CYB-3 is asymmetrically distributed such that the somatic precursor receives more than its germline precursor sister cell. This asymmetry is abolished in *par-4* mutant embryos, where all blastomeres contain equivalent amounts of CYB-3.

## Reagents

Antibody: Mab F2F4. Strain KK184 *par-4*(*it47ts*) V.
